# Use of the Wrapsody™ Cell-Impermeable Endoprosthesis for the treatment of pelvic congestion syndrome due to nutcracker syndrome

**DOI:** 10.1186/s42155-026-00748-x

**Published:** 2026-08-28

**Authors:** Elene Tkabladze, Karl Julius Büchner

**Affiliations:** Gefäßzentrum Am Ev. Krankenhaus Königin Elisabeth Herzberge gGmbH, Herzbergstraße 79, Berlin, 10365 Germany

**Keywords:** Nutcracker syndrome, Pelvic congestion syndrome, Wrapsody™ Cell-Impermeable Endoprosthesis

## Abstract

Endovascular stenting is a preferred treatment approach for the management of pelvic congestion syndrome (PCS) secondary to nutcracker syndrome. However, there is no consensus on which stenting device is advantageous for patients. In this report, we summarize off-label use of the Wrapsody™ Cell-Impermeable Endoprosthesis (CIE) for this condition. A 30-year-old woman presented with chronic left flank and lower abdominal pain, progressively worsening since childbirth. Imaging demonstrated compression of the left renal vein between the aorta and superior mesenteric artery with reflux into the ovarian vein. Endovascular treatment was performed using a 14 × 40 mm CIE. The procedure was technically successful and symptomatic resolution was achieved without complications. Follow-up over 2 years confirmed persistent device patency and absence of venous congestion. This case highlights that the CIE, a device originally designed for restoration of hemodialysis access, is an effective endovascular option for managing PCS due to nutcracker syndrome.

**Graphical Abstract:**

**Figure:**
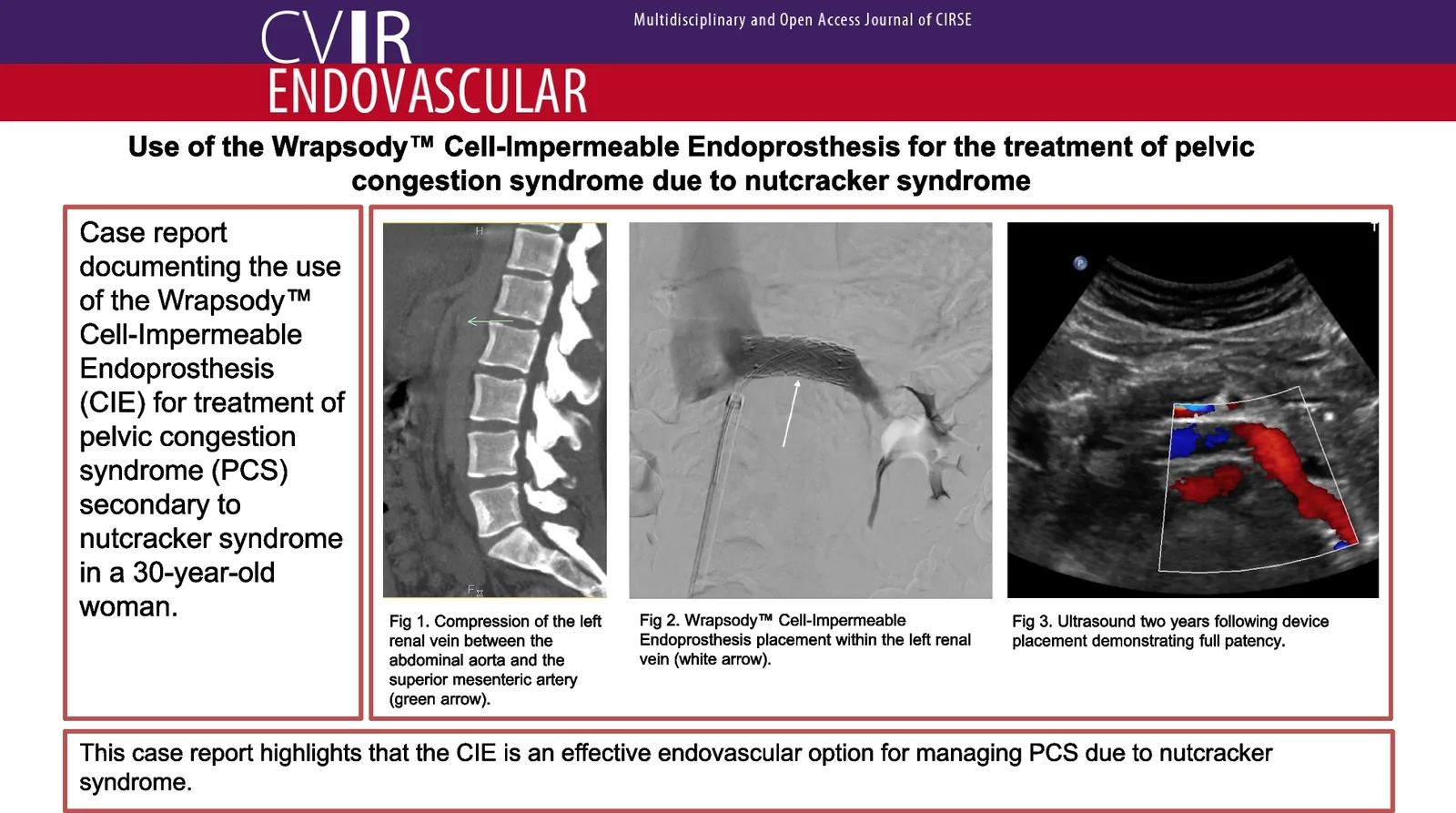

## Introduction

Pelvic congestion syndrome (PCS) is a gynecological condition characterized by chronic pelvic pain. Although first described in 1831, PCS is a rare condition that remains poorly understood [1] largely due to its generalized symptomatology. PCS mainly affects premenopausal multiparous women [2]. The pelvic pain characteristic of PCS is defined as persistent or intermittent pain that is present for more than 6 months that is unrelated to menstruation or pregnancy [3]. Factors that may contribute to PCS include primary venous insufficiency of the ovarian veins, reflux of ovarian veins, and compression of the left iliac common vein (May-Thurner syndrome) or the left renal vein (nutcracker syndrome) [4].

In recent years, growing recognition of venous insufficiency in the pelvic and retroperitoneal regions as a potential cause of chronic pelvic pain has helped vascular specialists diagnose and treat the condition. However, differentiating PCS from other gynecologic, gastrointestinal, or musculoskeletal disorders is challenging due to overlapping clinical features, which can delay appropriate management [1]. Awareness of these vascular etiologies is essential for timely diagnosis and the selection of minimally invasive endovascular interventions that can significantly improve patient outcomes.

Treatment options for PCS due to nutcracker syndrome range from open surgery to minimally invasive procedures. Endovascular stenting is often the preferred approach; however, there is no consensus on the stenting device used. This case report describes the off-label use of the Wrapsody™ Cell-Impermeable Endoprosthesis (CIE), originally designed for restoring vascular access in patients on hemodialysis who experience stenosis/occlusion in their venous outflow circuit [5], as a treatment option for PCS due to nutcracker syndrome.

## Case report

A 30-year-old married, G2P2 woman presented with recurrent pain in the left flank and lower abdomen. The patient’s pain was described as a cramping spasm that had progressively worsened over the past several years. The pain began following the birth of her first child in 2015. Initially mild, the discomfort increased in intensity after the birth of her second child in 2019. The pain was mainly localized to the left flank and did not radiate to her back. The pain worsened after prolonged sitting and standing and occurred mostly in the evening. No gastrointestinal or systemic symptoms were reported. The patient is a non-smoker and drinks alcohol occasionally. Her medical history included use of oral contraceptives prior to her first pregnancy and isotretinoin treatment for acne starting in 2021. She also underwent sinus surgery for nasal polyps in 2011. On physical examination, the patient appeared in good general health. Vital signs were within normal limits, her blood pressure was 120/80 mmHg, heart rate 66 bpm, and temperature 37.2 °C. Cardiovascular examination revealed normal heart sounds without murmurs. Her abdomen was soft, not tender, and showed no signs of organomegaly. Gynecological examination was unremarkable. Laboratory tests for liver, pancreatic, and renal function, as well as blood count, all returned within normal limits. Additionally, an antinuclear antibody (ANA) test was performed, which showed an ANA titer of 1:80, which is considered a low-positive titer. About 10–20% of healthy people, especially women and older adults, can have low-titer positive ANA. Urine exam showed proteinuria and microhematuria, consistent with nutcracker syndrome as compression of the left renal vein can increase intrarenal venous pressure, leading to rupture of small intrarenal veins and leakage of red blood cells and protein into the urine. Urine specific gravity was minimally decreased.

**Fig. 1:**
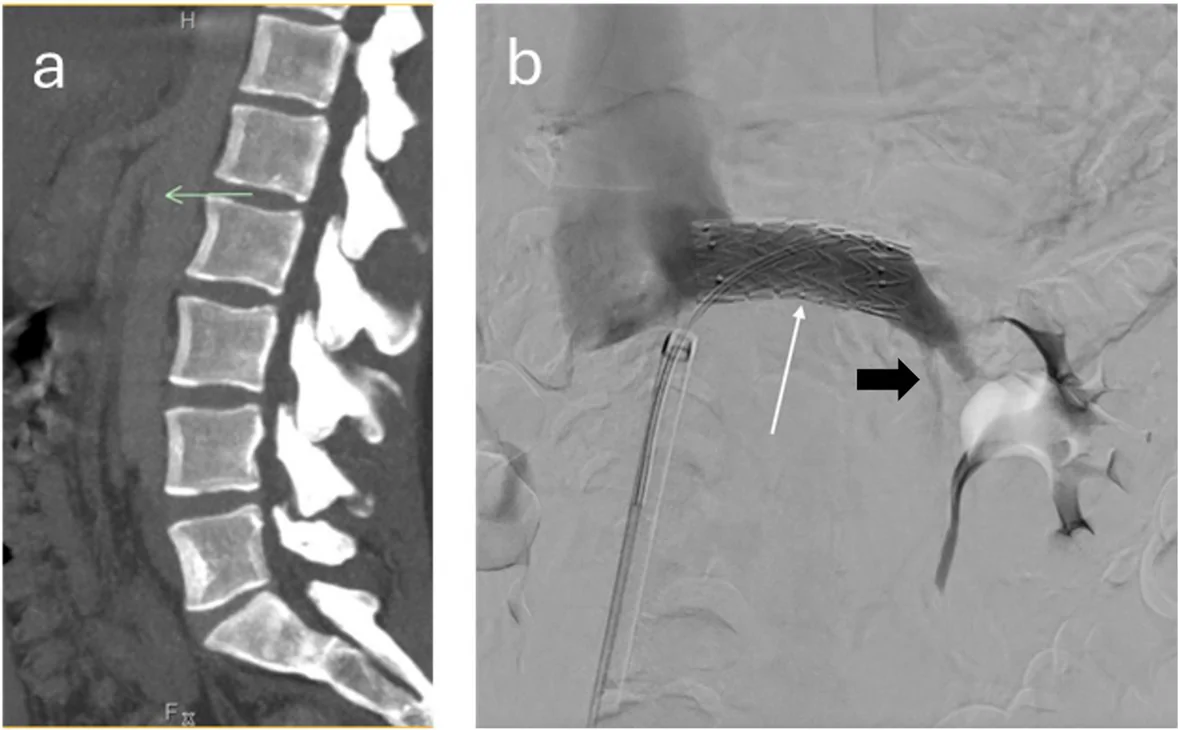
Compression of the left renal vein between the abdominal aorta and the superior mesenteric artery, green arrow (**a**); Wrapsody™ Cell-Impermeable Endoprosthesis placement within the left renal vein, white arrow, black arrow denotes ovarian vein (**b**)

**Fig. 2:**
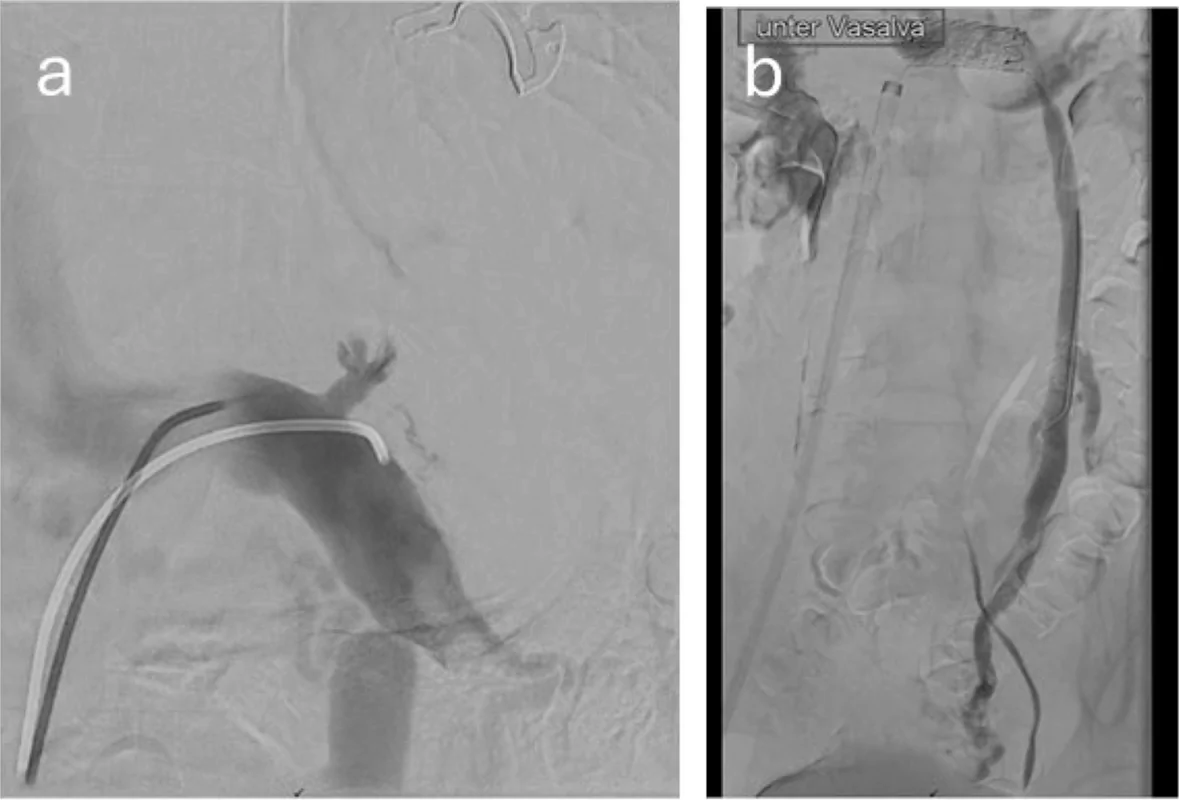
Angiography before stent placement showing a prestenotically dilated vein (**a**); under Valsalva maneuver, contrast reflux into the ovarian vein is observed (**b**)

Reno-caval pressure gradient measurement and an intravascular ultrasound were not performed as part of our institutional diagnostic protocol. Diagnosis was established based on the patient's clinical presentation together with ultrasound, computed tomography, magnetic resonance imaging, and venographic findings demonstrating significant left renal vein compression with ovarian vein reflux. Initial imaging with ultrasound showed an enlarged left kidney with a dilated appearance, consistent with venous congestion, while the kidney itself showed no evidence of hydronephrosis. The left renal vein appeared congested just before it passed between the aorta and the superior mesenteric artery, without evidence of thrombosis. Computed tomography imaging showed evidence of pre-stenotic dilatation of the left renal vein, consistent with impaired outflow. Interestingly, the vein was not visualized at the point where it should have crossed between these vessels, suggesting possible compression. Magnetic resonance imaging was performed to further assess the vascular anatomy. It confirmed the ultrasound findings and showed retrograde flow into the left ovarian vein. Computed tomography imaging revealed persistent dilation of both the left renal and ovarian veins with caliber change of the left renal vein between the abdominal aorta and the superior mesenteric artery (left renal vein diameter ratio of 4.5 and an aortomesenteric angle of 13°), a hallmark of nutcracker syndrome (Figs. 1 and 2).

To confirm our findings and planned treatment approach, we performed phlebography of both the left renal and ovarian veins. Based on phlebography findings, we treated the left renal vein with a 14 × 40 mm CIE (Merit Medical Systems, Inc., South Jordan, UT, USA). The device has a unique cell-impermeable middle layer with maximal radial resistive force typically used for dialysis shunts. For this case, the device was considered suitable for treating renal vein compression due to its structural integrity.

The procedure was performed via standard venous access. Activated clotting time was not measured during this procedure. Intraprocedural anticoagulation was achieved with aspirin 500 mg and heparin 3000 IU. The stenotic left renal vein segment was predilated sequentially with 10 × 40 mm and 12 × 40 mm balloons, followed by further dilatation up to 20% oversizing relative to the target vein diameter, and post-dilatation with a 14 × 20 mm balloon to optimize stent apposition. We estimated the reference vessel diameter to be approximately 11 mm and considered deploying either a 12 mm or a 14 mm device. Ultimately, we selected a 14 mm device to reduce the risk of stent migration, as the 12 mm balloon appeared undersized within the vessel and reached full expansion rapidly, suggesting that it was not providing adequate apposition to the vessel wall. A CIE was deployed across the aortomesenteric portion of the left renal vein, carefully positioned to preserve the ostia of the left ovarian and adrenal veins, which remained patent. Following deployment of the device, retrograde flow was minimized, the ovarian vein decreased in size, and no spontaneous reflux was observed. The device was carefully positioned to preserve the ovarian vein and other tributaries (Fig. 2a). No procedural complications were observed. Post-procedurally, the patient was initiated on a lifelong antiplatelet regimen of aspirin (100 mg daily) based on our institutional protocol. We acknowledge that this regimen may differ from other published practices. However, there is currently no high-quality evidence or consensus guideline defining the optimal antithrombotic regimen following covered venous stent-graft implantation, and clinical practice remains heterogeneous across institutions. No systemic anticoagulation was administered. The patient reported complete symptom resolution at follow-up.

**Fig. 3:**
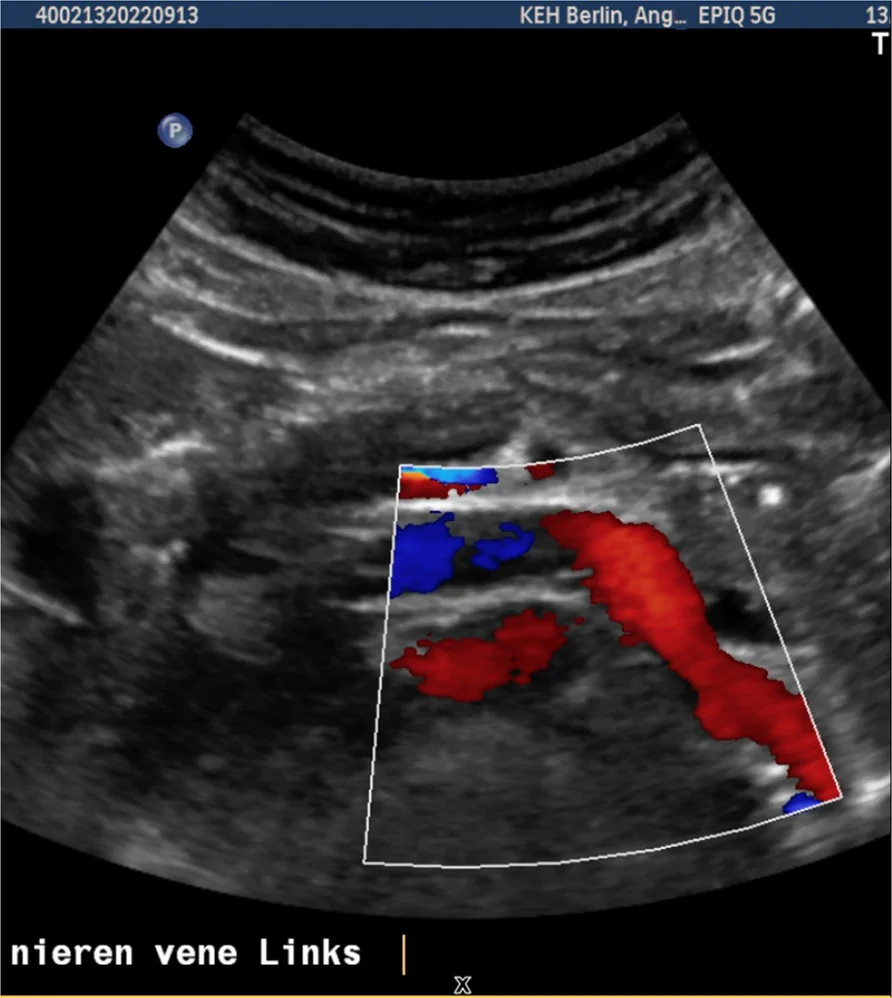
Ultrasound 2 years following device placement demonstrating full patency

The patient was evaluated clinically and with duplex ultrasound imaging at 2, 4, 10, 12, and 24 months after the procedure to confirm device patency and evaluate venous flow. Although no standardized pain assessment (e.g., Visual Analog Scale) was provided to the patient, she did report complete resolution of pain in the flank and pelvic regions. Laboratory evaluations during the follow-up period indicated resolution of microhematuria and proteinuria. The patient’s renal function remained stable throughout the follow-up period. Follow-up ultrasound imaging confirmed patency of the device with stable blood flow and no evidence of flow acceleration, indicating the absence of a hemodynamically significant stenosis. Two years after the procedure, ultrasound imaging showed no recurrence of congestion or reflux (Fig. 3). Given the stable imaging findings and the patient's favorable clinical outcome, magnetic resonance imaging was not considered necessary during follow-up.

## Discussion

While conventional surgical options, including left renal vein transposition and renal autotransplantation, are effective, they carry significant perioperative morbidity, prolonged hospitalization, and extended recovery [6, 7]. Based on our experience, an endovenous approach is the preferred initial treatment for PCS due to nutcracker syndrome as it is minimally invasive, safe, and provides long-term symptomatic resolution. Moreover, there is growing recognition of the clinical utility of minimally invasive endovascular interventions for this patient population. High technical success rates have been reported in patients following an endovascular intervention. Furthermore, patency rates associated with stent placement are generally high, with one study reporting a 2-year primary patency rate of 85.2% [8]. However, stent migration remains a notable risk, reported in 4.9–7.3% of cases, and can lead to life-threatening complications such as embolization to the inferior vena cava, right heart, or pulmonary arteries, resulting in arrhythmias, heart failure, or pulmonary infarction [9–11].

Given the non-negligible risk associated with stent migration, research comparing different devices is warranted to explore how this complication can be mitigated. It is our opinion that certain practical design advantages of the WRAPSODY device may contribute to its ability to reduce risk of migration in the treatment of left renal vein compression. To our knowledge, this is the first case report summarizing use of the WRAPSODY CIE for endovascular treatment of PCS due to nutcracker syndrome. The specific design features prompting use of this device included its high radial resistive force, low foreshortening, and its tri-layered design. It is our opinion that these features may offer practical advantages that may provide durable vessel scaffolding. However, we acknowledge that a focused clinical study is necessary to explore this relative to outcomes associated with other devices. Additionally, while the patient achieved symptomatic relief over the 2-year follow-up, additional research is needed to evaluate the device’s long-term efficacy, beyond 2 years, in alleviating chronic pelvic pain and other complications associated with PCS due to nutcracker syndrome.

## Conclusion

Endovascular stenting is often a preferred intervention for PCS due to nutcracker syndrome. In the absence of consensus on a stenting device to use in this patient population, we summarize a single case involving the successful off-label use of the WRAPSODY CIE. The safe and durable symptomatic relief for PCS due to nutcracker syndrome in this patient warrants further investigation of this device.

## Data Availability

Data sharing is not applicable to this article as no datasets were generated or analyzed during the current study.

## Abbreviations

ANA: Antinuclear antibody
CIE: Cell-Impermeable Endoprosthesis
PCS: Pelvic congestion syndrome
